# Green synthesis of silver nanoparticles using *Solanum mammosum* L. (Solanaceae) fruit extract and their larvicidal activity against *Aedes aegypti* L. (Diptera: Culicidae)

**DOI:** 10.1371/journal.pone.0224109

**Published:** 2019-10-31

**Authors:** Fernanda Pilaquinga, Bianca Morejón, Danny Ganchala, Jeroni Morey, Neus Piña, Alexis Debut, Marco Neira

**Affiliations:** 1 Department of Chemistry, University of the Balearic Islands, Palma de Mallorca, Balearic Islands, Spain; 2 Laboratory of Nanotechnology, Department of Exact and Natural Sciences, Pontificia Universidad Católica del Ecuador, Quito, Ecuador; 3 Center for Research on Health in Latin America, Department of Exact and Natural Sciences, Pontificia Universidad Católica del Ecuador, Quito, Ecuador; 4 Centro de Nanociencia y Nanotecnología, Universidad de las Fuerzas Armadas ESPE, Sangolquí, Ecuador; Australian Red Cross Blood Service, AUSTRALIA

## Abstract

The family of mosquitoes (Diptera: Culicidae) contains several species of major public health relevance due to their role as vectors of human disease. One of these species, *Aedes aegypti*, is responsible for the transmission of some of the most important vector-borne viruses affecting humankind, including dengue fever, chikungunya and Zika. Traditionally, control of *Ae*. *aegypti* and other arthropod species has relied on the use of a relatively small diversity of chemical insecticides. However, widespread and intensive use of these substances has caused significant adverse environmental effects and has contributed to the appearance of pesticide-resistant populations in an increasing number of locations around the world, thereby dramatically reducing their efficiency. Therefore, it becomes urgent to develop novel alternative tools for vector control. In that context, our study aimed at evaluating the insecticidal activity against *Ae*. *aegypti* of aqueous extracts obtained from the fruits of *Solanum mammosum* L., as well as silver nanoparticles synthesized using aqueous extracts from this plant species (SmAgNPs). To perform the test, third instar *Ae*. *aegypti* larvae were exposed to increasing concentrations of plant extract and SmAgNPs for 24 h. Our results suggest that both the aqueous extract and SmAgNPs were toxic to the larvae, with SmAgNPs displaying a much higher level of toxicity than the extract alone, as reflected in their LC_50_ values (0.06 ppm vs 1631.27 ppm, respectively). These results suggest that both *S*. *mammosum* extracts and SmAgNPs exhibit noteworthy larvicidal activity, and should be further explored as potential source of alternative tools in the fight against insect vectors of human disease.

## Introduction

*Aedes aegypti* continues to be one of the most important vectors of human arboviral disease world-wide, including yellow fever, dengue, chikungunya and Zika [1]. Recently, global trends such as the massive movement of people and goods across international borders, disorganized urbanization and ecological disruption have facilitated the emergence and re-emergence of infectious diseases vectored by *Ae*. *aegypti*, through the dissemination and establishment of this species along the tropical and subtropical regions of the planet [2,3].

Since no commercial vaccines exist for most diseases spread by *Ae*. *aegypti* (with the notable exception of yellow fever [4]), mosquito control remains the key component of all prevention and control campaigns, which usually rely on strategies such as pesticide spraying, the use of biological control agents and environmental management, among others [5].

Following the discovery of the first synthetic insecticidal molecules in the 1940´s, most mosquito control programs around the world have become heavily dependent on the use of increasing amounts of chemical pesticides, including organochlorines, organophosphates, carbamates and pyrethroids, among other [6,7]. While the adult stages of medically important mosquitoes are usually controlled by spraying pesticides around the areas where these species forage and rest, the aquatic larval stages are controlled by either draining breeding sites or rendering them toxic to mosquito larvae through the use of larvicides such as organophosphates, growth inhibitors or bacterial insecticides.

This marked dependence on chemical spraying has caused alarm in the international scientific community due to multiple studies revealing the negative effects that long term use of these products has on non-target organisms such as humans, wildlife, fish and arthropod species [8–11]. Furthermore, an equally alarming side effect is the appearance of pesticide-resistant insect populations around the world, including Central and South America [12–16]. Therefore the development of novel, environmentally friendly and efficient tools for mosquito control is of paramount importance in order to ensure our future capacity to prevent and control the diseases transmitted by these insects.

Because of their vast biological diversity, plants represent an important source of potential novel insecticides. Furthermore, the natural complexity of plant-derived insecticidal extracts can provide an additional advantage in the fight against pesticide resistance: since these extracts often contain mixtures of several chemical compounds that can act synergistically on different molecular targets within the insect, the probability of survival of individuals displaying resistance mechanisms against any one of those chemicals is greatly reduced [17,18].

Another novel technology with clear potential applications in the field of insect control is nanotechnology. Specifically, the synthesis of silver nanoparticles (AgNPs) incorporating extracts of plants displaying insecticidal activity has been shown to create larvicidal compounds which are efficient against mosquitoes at very low concentrations [19–22]. Additionally, the synthesis of such plant-derived AgNPs does not require high pressure, energy, temperature or the use of highly toxic chemicals; therefore, it is considered cost-effective, environmentally friendly and time-efficient [22,23].

The Solanaceae are a group of plants comprising approximately 2 700 species, grouped in 98 different genera. This botanical family has a cosmopolitan distribution and includes several species of major economical relevance, including the potatoes (*Solanum tuberosum*) and tomatoes (*S*. *lycopersicum*) [24]. In Ecuador, there are 338 plant species belonging to this family [24] and several of them have been reported to be used as insecticides by local communities [25]. Among these, *S*. *mammosum* stands out due to the high number of communities reporting the use of this species as an insecticide [25]. *S*. *mammosum* is a species native to Ecuador and is found in the Coastal and Andean region of the country, between 0 and 500 meters above sea level (m.a.s.l.) [24]. Both the species name (*mammosum)* and its common name (“nipple-fruit nightshade”) refer to the peculiar nipple-like protrusions at the base of its fruits [26], which are the part of the plant used by the local populations to kill small arthropods [25].

Here, we report the methodology for synthesizing functionalized AgNPs using aqueous extracts of *S*. *mammosum* fruit. Additionally, we present the results of bio-assays evaluating the larvicidal activity of these aqueous extracts and AgNPs against *Ae*. *aegypti* 3^rd^ instar larvae.

## Materials and methods

### Collection of plant specimens

**Fig 1:**
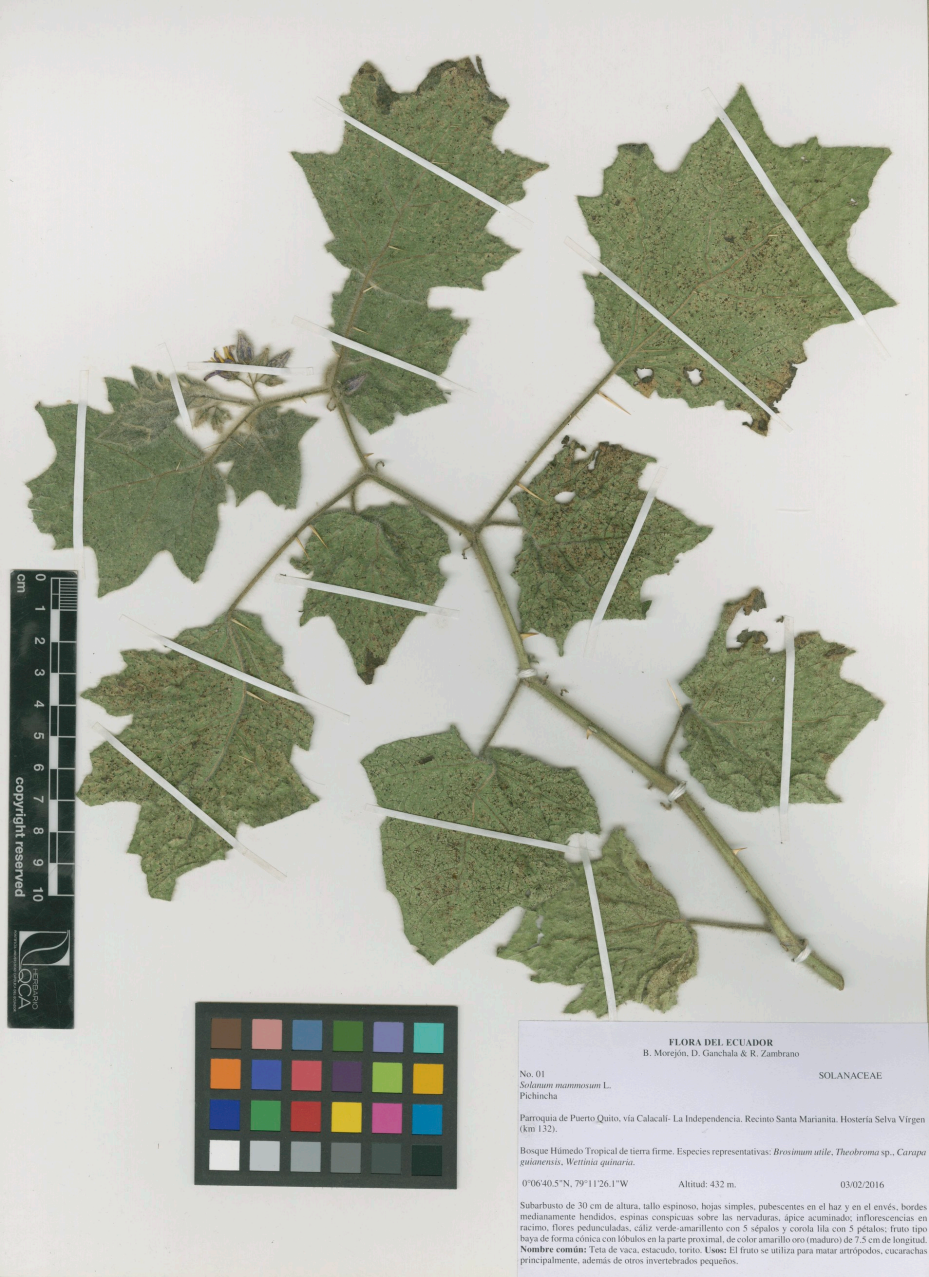
*Solanum mammosum* (L.) botanical voucher. This reference specimen is deposited at the Center for Research on Health in Latin America (CISeAL) in Quito, Ecuador.

We collected fresh and ripe *S*. *mammosum* fruits in the Marianitas precinct (located at 0°05’46.3”N, 79°07’33.5”O; altitude: 135 m.a.s.l.), near the town of Puerto Quito, approximately 140 km north-west of Ecuador´s capital city, Quito. A botanical voucher (Fig 1) was prepared and deposited for reference at the Center for Research on Health in Latin America (CISeAL).

### Preparation of aqueous extract

Fruits were washed several times with deionized water to remove any impurity. To obtain the extract the fruits were dried in an oven at 25° C for five days, ground in an electric mill, and stored in a refrigerator (4°C) until used. One gram of the pre-ground dried fruit was mixed with 20mL deionized water in an Erlenmeyer flask. This mixture was brought to room temperature, with constant agitation, for one hour. Before being used for nanoparticle synthesis, the aqueous extract was vacuum-filtered using Whatman^TM^ 41 filter paper (GE Healthcare), which has a pore size of 20μm. The filtered extract was stored at 4° C until used.

### Synthesis of *S*. *mammosum-*coated AgNPs

To obtain AgNPs coated with *S*. *mammosum* extract (SmAgNPs), two mL *S*. *mammosum* extract were added dropwise to 20mL of a 1mM AgNO_3_ solution. The pH of this mixture was adjusted to 9 using a 1% NaOH solution, and the mixture was subsequently stirred for 30 minutes at 35 (± 1)°C. Once the mixture changed color from light yellow to brown (indicating the reduction of Ag^+^ to Ag) the solution was exposed to UV light (366 nm wavelength) for one hour.

### Characterization of SmAgNPs

Surface Plasmon Resonance (SPR) bands of the synthesized SmAgNPs were characterized using a Cary 60 UV–Vis spectrophotometer (Agilent Technologies), within the range of 300 to 800nm. The elemental analysis was obtained by scanning electron microscopy/energy dispersive x-ray spectrometry (SEM-EDX) which was performed on a FEG-SEM chamber using a Bruker X-Flash 6|30 detector, with a 123 eV resolution at Mn Kα. The morphological analysis was elaborated using a Tescan Mira 3 microscope equipped with a Schottky Field Emission Gun (Schottky FEG-SEM) which reaches a resolution of 1.2 nm at 30 keV. Transmission electron microscopy (TEM) micrographs were obtained using a Tecnai G20 Spirit Twin from (FEI). Dynamic light Scattering (DLS) measurements for determining the average size and size distribution of the silver nanoparticles were carried out using the LB-550 DLS Nanoparticle Size Analyzer (Horiba). All DLS measurements were performed at a fixed temperature of 25 ºC. X-ray Diffraction (XRD) was performed using an Empyrean diffractometer from PANalytical operating in a θ-2θ configuration (Bragg-Brentano geometry) and equipped with a Cu X-ray tube (Kα radiation λ = 1.54056 Å) operating at 40 kV and 40 mV.

### Mosquito culture

The strain of *Ae*. *aegypti* used in our experiments was first collected in 2015 in the city of Puerto Francisco de Orellana, Orellana Province, Ecuador, and has since been continuously maintained at CISeAL under standard insectary conditions (28 ±1° C temperature, 80 ± 10% relative humidity, 12-h light/12-h darkness photoperiod) [27]. *Ae*. *aegypti* eggs were hatched in distilled water that had been previously boiled to reduce oxygen content, and cooled down to room temperature. Larvae were fed on finely ground fish food flakes, following the feeding regime developed by [28]. The larvae were maintained at the aforementioned standard insectary conditions until reaching the 3^rd^ instar, when they were used for experimentation.

### Larvicidal assays

Tests aimed at evaluating the larvicidal activity of *S*. *mammosum* extracts and SmAgNPs were performed following the protocol proposed by [29], with some modifications described in [30].

The concentration ranges of *S*. *mammosum* extracts and SmAgNPs used in the bioassays were established in several preliminary range-finding tests (data not shown). Concentrations of *S*. *mammosum* extract used in bioassays were 1500, 3000, 4500, and 6000 ppm. Concentrations of SmAgNPs used in bioassays were 0.05, 0.06, 0.07 and 0.08 ppm.

For each bioassay, we created a set of four experimental groups (one for each concentration tested) and a control group. For each group, the basic testing unit (i.e. each technical replicate) was a plastic beaker where 25 third-instar *Ae*. *aegypti* larvae were placed in 200 mL of the test solution at the chosen concentration (or distilled water, in the case of the control group). Each group contained four technical replicates, for a total of 500 larvae per bioassay. The entire bioassay was repeated five times.

During the bioassay period, larvae were kept at the aforementioned standard insectary conditions and they were not fed. After 24 hours of exposure, we recorded mortality. Individuals were considered to be dead if, after stimulation by touch, they didn´t move at all or moved sluggishly and were unable to rise towards the surface of the water [29].

### Data analysis

Average larval mortality data from the five bioassay replicates were subjected to dose-response regressions using a log-probit model [31] in order to calculate LC_50_ and LC_90_ values. Calculations were performed using the R-software for statistical computing [32] and codes of the MASS package [33].

## Results and discussion

### Results

#### Characterization of synthesized SmAgNPs

**Fig 2:**
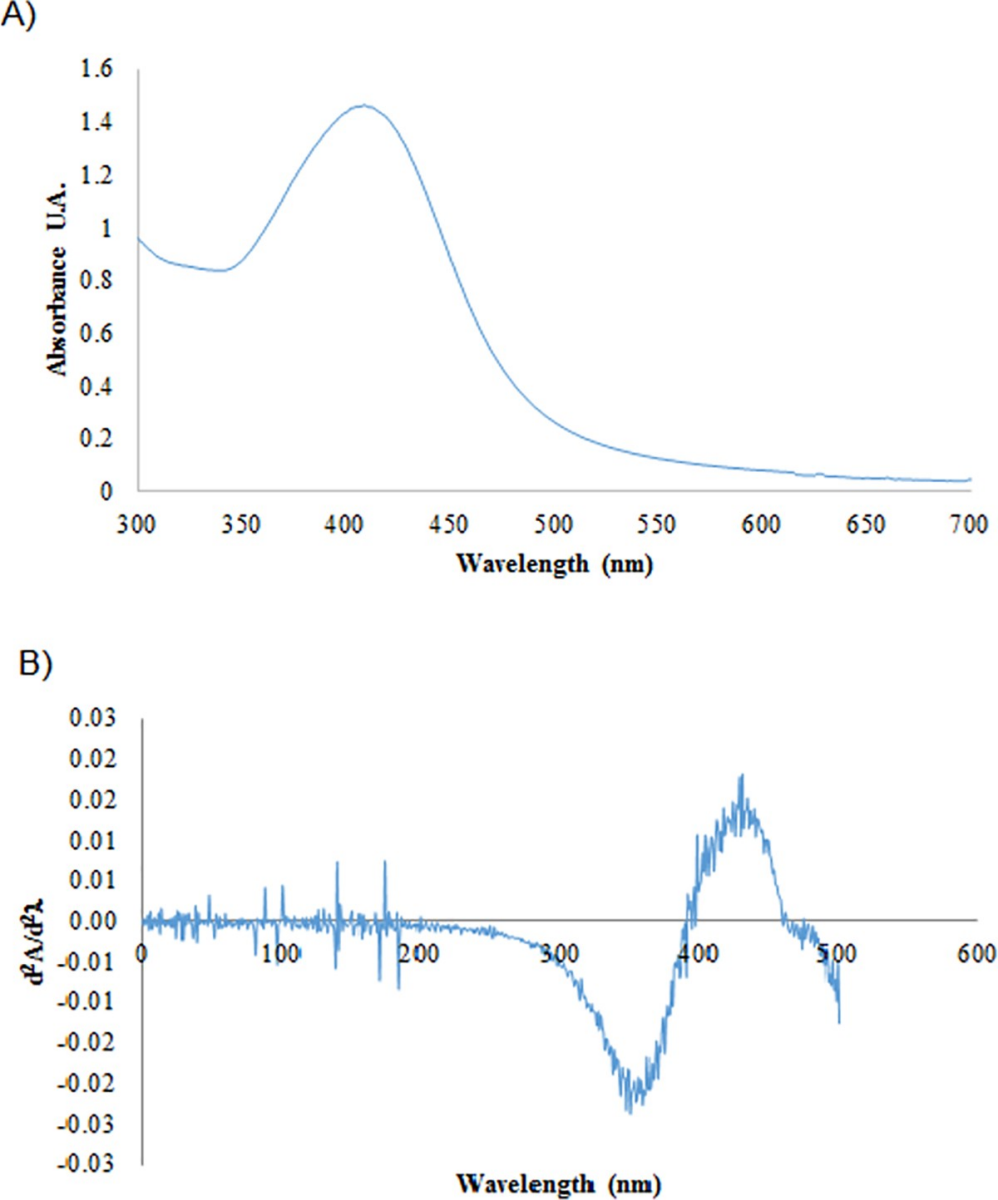
UV-Vis absorption spectrum of SmAgNPs. A) UV-Vis absorption spectra (AgNPs 1mM 1:10 v/v using deionized water). B) Second derivative UV-Vis spectrum.

The optimized formation of SmAgNPs monitored by UV-Vis spectroscopy shows a maximum absorption band at 411.5 nm (Fig 2A), corresponding to the surface plasmon resonance SPR. This result was confirmed by second derivative UV-Vis spectrum (Fig 2B).

**Fig 3:**
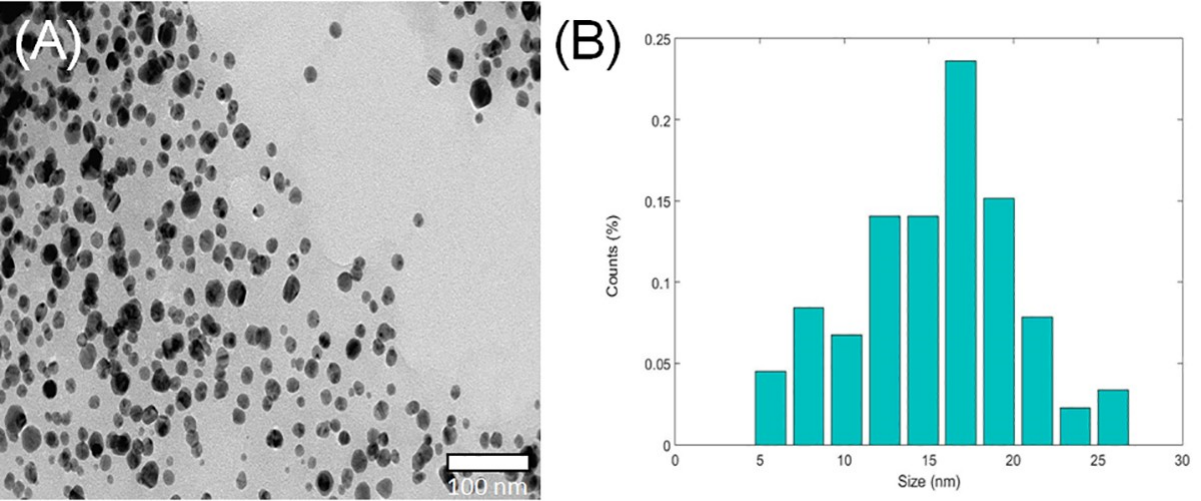
TEM micrograph of SmAgNPs. (A) TEM 80 kV and (B) size distribution histogram.

According to Pradeep's description [34], the average wavelength obtained corresponds to an average nanoparticle size of 10 to 14 nm. This result agrees with the TEM analysis performed (Fig 3). A normal distribution fit with a 95% confidence interval gives an average value of 15.3±4.8 nm. The nanoparticles were found to be highly dispersed in solution, confirming that the plant extract acts as a stabilizing agent as well as a reducing agent.

The *Z*-average calculated from the DLS data was 104.9 ± 59.9 nm. The difference between TEM and DLS analysis is related to the electrical layer adhered to the nanoparticle surface [35]. This difference suggests that the organic core-shell of *S*. *mammosum* is over the nanoparticle, thus increasing the thickness of the surface layer. Nanoparticle size distribution can be articulated through the polydispersity index (PDI). PDI for our SmAgNPs was of 0.38, a value that indicates a moderately disperse distribution.

**Fig 4:**
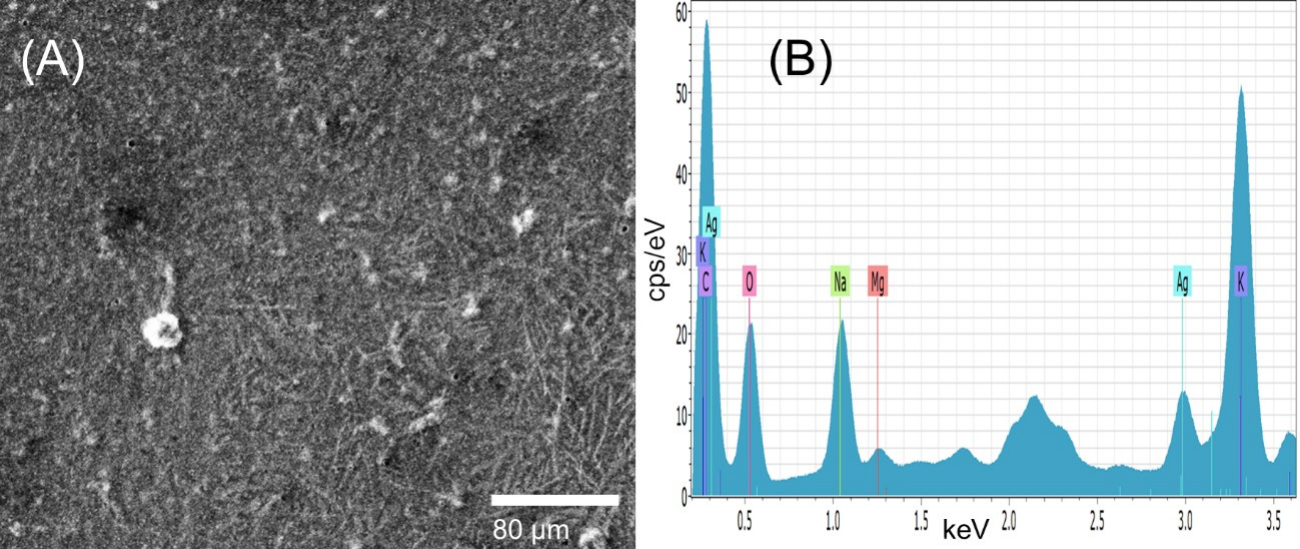
SEM micrograph of SmAgNPs 1M. (A) SEM 25 kV and (B) EDX spectrum.

The SEM micrograph in Fig 4A shows the dispersion as bright points of SmAgNPs powder. For EDX (Fig 4B), the material was fixed in a plate previously covered with two layers of double-coated carbon conductive tape. In order to avoid biased determinations of the chemical compositions of the samples due to their inhomogeneity, we have averaged the spectra obtained from a 25-point grid on a total area of at least 0.2 mm^2^. The presence of each element is denoted by the normalized weight percentage, which is the percentage in weight assuming that the chosen elements represent the total composition of the sample. We have refrained from calculating the presence of C and O, for which the abundance in porous samples is higher than 50%. The EDX spectrum presented in Fig 4B shows the three characteristic peaks of silver under 3keV due to surface plasmon resonance. Silver abundance is about 26.9% normalized mass percent. The other elements are Na (22.7%), Mg (4.6%), S (6.8%), K (36.4%) and Ca (2.5%).

**Fig 5:**
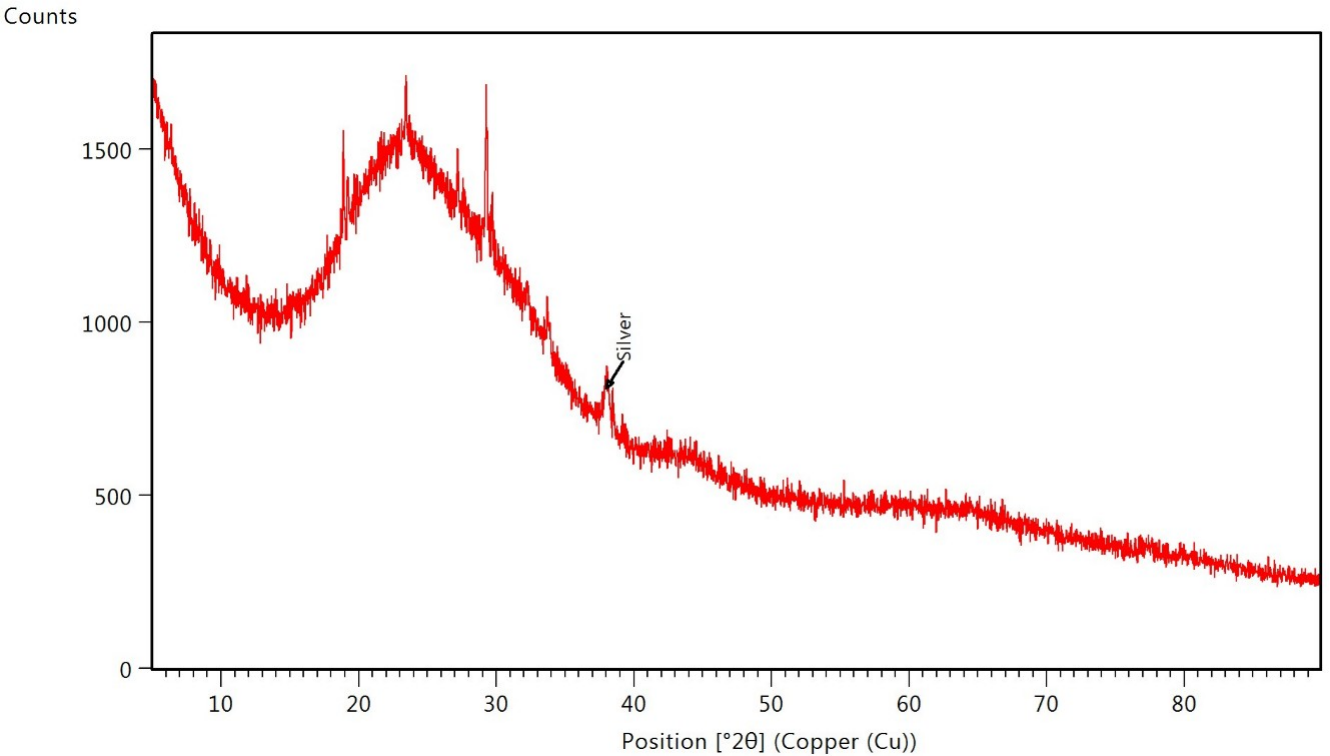
X-ray diffraction pattern of SmAgNPs. Data shown correspond to the average of six measurements (5° to 90°).

Samples used for XRD were dried on a microscope slide at 40°C to avoid any organic degradation. Six measurements from 5° to 90° (θ-2θ) were performed, and were used to obtain an average value. The resulting XRD pattern (Fig 5) confirms the crystalline nature of SmAgNPs. The Bragg reflection peak at 38.04° coincides with the cubic phase of silver (Inorganic Crystal Structure Database—ICSD: 180875). The (111) lattice parameter and highest intensity plane is well matched and agrees with other reported patterns [35–37]. Further peaks can be observed around 19° and 27°-29°, which probably correspond to silver nitride and silver nitrate compounds mixed with the *S*. *mammosum* extract. This suggests that SmAgNPs are not the only inorganic compounds created, and that a competitive mechanism is part of the chemical synthesis process. Other observed peaks are attributable to impurities coming from the extract.

To estimate the approximate size of SmAgNPs, we applied Debye Scherrer’s equation. At highest reflection peak (FWHM = 0.463°), this equation provides an estimated size of approximately 19.0 nm. This value agrees with the measurements obtained by TEM.

### Larvicidal activity of aqueous extract and synthesized SmAgNPs

Table 1 shows the results of bioassays performed using both the plant aqueous extract and SmAgNPs. Mortality rates were dose-dependent in specimens exposed to both types of treatment. Control groups showed no mortality.

**Table 1:** Larvicidal activity of aqueous extracts and silver nanoparticles synthesized with aqueous fruit extract of *S*. *mammosum* against *Ae*. *aegypti* third instar larvae.

Treatment	Dose (ppm)	Larval mortality ^a^ ±SD
**Aqueous extract**	0 (control)	0 ± 0.0
	1500	49.2 ± 2.9
	3000	71.2 ± 4.0
	4500	87.8 ± 3.1
	6000	97.0 ± 2.7
**SmAgNPs**	0 (control)	0 ± 0.0
	0.05	19.0 ± 1.2
	0.06	51.0 ± 2.9
	0.07	72.0 ± 3.3
	0.08	93.4 ± 3.6

^a^ Values shown represent the mean of five replicates.

SD: standard deviation.

The LC_50_ and LC_90_ values of *S*. *mammosum* aqueous extract and SmAgNPs are presented in Table 2. For *S*. *mammosum* extract, LC_50_ and LC_90_ values corresponded to 1 631.27 ppm and 4 756.20 ppm, respectively. For SmAgNPs, LC_50_ and LC_90_ values corresponded to 0.06 ppm and 0.08 ppm, respectively. As is evident, the toxicity of SmAgNPs to *Ae*. *aegypti* larvae is exponentially higher than the toxicity of *S*. *mammosum* extract by itself. For both the aqueous extract and SmAgNPs, χ^2^ value was not significant at p ≤ 0.05 level.

**Table 2:** Probit values of plant aqueous extracts and silver nanoparticles synthesized with aqueous fruit extract of *S*. *mammosum* against *Ae*. *aegypti* third instar larvae.

Treatment	LC_50_ (ppm)	95% Fiducial Limits (LCL-UCL)			LC_90_ (ppm)	95% Fiducial Limits (LCL-UCL)			χ^2^
	**± SE**				**± SE**				
**Aqueous extract**	1631.27 ± 1.09	1311.40	-	1901.25	4756.20 ± 1.09	4081.70	-	5913.71	3.76 ^NS^
**SmAgNPs**	0.06 ± 1.02	0.05	-	0.62	0.08 ± 1.03	0.07	-	0.08	1.76 ^NS^

LC_50_: lethal concentration that kills 50% of the exposed larvae

LC_90_: lethal concentration that kills 90% of the exposed larvae

LCL: lower confidence limit

UCL: upper confidence limit

^NS^: not significant at p ≤ 0.05 level

χ^2^: Chi-square test.

## Discussion

Our results show that both the aqueous extract of *S*. *mammosum* fruit and SmAgNPs are toxic to third instar *Ae*. *aegypti* larvae. However, the toxicity of SmAgNPs is at least 20 000 fold higher than that of the aqueous extract alone, as evidenced by the difference in LC_50_ values (Table 2).

Several studies have reported the larvicidal activity of plants of the Solanaceae family against *Ae*. *aegypti*. Chowdhury and collaborators evaluated the chloroform: methanol extract of *S*. *villosum* fruit, reporting an LC_50_ of 11.67 ppm [38]; Raghavendra and collaborators evaluated the aqueous extract of *S*. *nigrum* fruit, reporting an LC_50_ of 359 ppm [39]. Mahesh-Kumar and collaborators tested the methanolic extract of *S*. *xanthocarpum* fruit, reporting an LC_50_ of 253.18 ppm [40]. Premalatha and collaborators tested the acetonic, chloroformic and methanolic extracts of *S*. *trilobatum* against L4 larvae, and obtained LC_50_ values of 125.67 ppm, 125.87 ppm and 125.43 ppm, respectively [41]. And finally, Patil and collaborators tested the dichloromethanic extract of *Cestrum nocturnum* leaves against L3 larvae, obtaining an LC_50_ of 30.12 ppm [42].

The LC_50_ value of the aqueous extract of *S*. *mammosum* fruit used in our study is higher than the LC_50_ values reported for other Solanaceae fruit extracts. However, it is important to consider the type of solvent used for the extraction. With the exception of the *S*. *nigrum* aqueous extraction [39], all of the extractions mentioned above were performed using organic compounds as solvents. It is therefore plausible that organic extractions produce solutions with a higher toxicity than aqueous extractions. When alkaloids are conformed as salts, they are soluble in water, but if they are conformed as free bases, they are soluble in non-polar organic solvents [43]; therefore, it is possible that the concentration of at least some potentially toxic compounds present in *Solanum* plants is lower in aqueous solutions than it would be in organic solutions. Furthermore, it is also plausible that organic solvents may present some level of toxicity by themselves, which would enhance any toxicity attributable to plant-derived chemicals.

Only two additional reports exist using AgNPs synthesized with nightshade species (i.e. species belonging to the *Solanum* genus) as potential mosquito larvicides: Rawani and collaborators used AgNPs synyetized from the aqueous extract of *S*. *nigrum* against third instar larvae of *Culex quinquefasciatus* and *Anopheles stephensi*, obtaining a LC_50_ of 2.44 and 1.54 ppm respectively [44]. And Murugan and collaborators used AgNPs obtained using aqueous extracts of *Datura metel* leaves against the larvae of *An stephensi*, reporting an LC_50_ of 4.288 ppm [45]. Therefore, to the best of our knowledge, the LC_50_ values obtained in our study are the lowest reported for any AgNPs synthetized using extracts of plants from the *Solanum* genus. Additionally, we are aware of a single study that explores the use of AgNPs syntetized from Solanaceae plants as *Ae*. *aegypti* larvicides: Govindarajan and collaborators reported on the use of AgNPs syntesized using an aqueous extract of *Nicandra physaloides* leaves, obtaining an LC_50_ of 13.61 ppm [46]. Therefore, the LC_50_ obtained in our work is also the lowest reported for AgNPs synthesized from any member of the Solanaceae family against *Ae*. *aegypti*.

It has been reported that species belonging to the Solanaceae family can present a prominent toxicity attributable to the presence of a wide repository of alkaloids, including tropane- alkaloids, glycoalkaloids, pirrolizidin and indol alkaloids, which are produced as a natural defense mechanism against insects, predators and infectious agents [47]. Alkaloids typical of Solanaceae plants, including α-tomatine, α-chaconine and α-solanine have been reported as displaying insecticidal activity against species of economic and medical importance [48,49]; therefore, it seems plausible that the lethal effect observed in the present study is, at least partially, due to the presence of these chemicals in the *S*. *mammosum* aqueous extract. Further research is required in order to isolate and characterize the specific chemicals responsible for the insecticidal action observed in *S*. *mammosum* extract.

The physiological basis for the high toxicity of plant-synthesized AgNPs remains an open question. It has been suggested that a key factor is their ability to permeate through the invertebrate exosqueleton and penetrate into the insect’s cells, where they bind macromolecules such as proteins and DNA, altering their structure and therefore their functionality [50]. Interestingly, it has also been reported that doses of plant-synthesized AgNPs which result lethal to several species of mosquito larvae, can have little or no effect on other non-target species, including other aquatic arthropod species and fish [46,51], suggesting that at least some mosquito species are particularly susceptible to the lethal effect of AgNPs. The reasons behind this phenomenon remain unknown, which highlights the need for further research aimed at establishing the biological effects of AgNPs in both target and non-target species.

## Conclusions

Our results suggest that the aqueous extract obtained from *S*. *mammosum* fruit is an effective larvicide against *Ae*. *aegypti*. Additionally, our data show that these fruit extracts can act as reducing agents for the synthesis of silver nanoparticles, and that said nanoparticles can kill *A*. *aegypti* larvae at significantly lower concentrations than the plant´s aqueous extract alone. In fact, the toxicity of SmAgNPs to *Ae*. *aegypti* larvae seems to be among the highest reported for AgNPs synthetized using any species belonging to the Solanaceae family.

Based on these results, we propose that *S*. *mammosum* has the potential to be a novel source of insecticides against insect species of public health relevance. However, further research is required to (a) identify and characterize the specific chemicals responsible for the observed insecticidal activity, (b) understand the exact biological mechanisms responsible for the lethal effects of *S*. *mammosum* extract and SmAgNPs, and (c) evaluate potential effects of these chemicals in the environment and in non-targets organisms. Once this information is available, it would be possible to establish whether any compounds derived from S. *mammosum* should be considered for further development as insecticides.
